# P-167. The Utility of Microbial Cell Free DNA in the Rapid Diagnosis of Acute Chagas Disease

**DOI:** 10.1093/ofid/ofaf695.391

**Published:** 2026-01-11

**Authors:** Anissa Amilhamja, Adam Shaikh, Kristin E Mondy, Michael A Kron, Niki Viradia, Omer E Beaird, Jaime Fergie

**Affiliations:** Driscoll Children’s Hospital, Corpus Christi, TX; Driscoll Children’s Hospital, Corpus Christi, TX; Dell Medical School at the University of Texas, Austin, Austin, Texas; Medical College wisconsin, Milwaukee, Wisconsin; Medical College of Wisconsin, MILWAUKEE, Wisconsin; University of California Los Angeles, Los Angeles, California; Infectious Disease, Driscoll Children's Hospital, Corpus Christi, Texas

## Abstract

**Background:**

Chagas disease (CD), caused by *Trypanosoma cruzi,* is a vector-borne infection with potentially severe cardiac and neurological complications during acute infection or reactivation. Endemic to Latin America, sporadic cases also occur in the United States. Diagnosis of acute CD is by microscopy of whole blood or tissue and/or a polymerase chain reaction (PCR) test, however, sensitivity of microscopy may be lower in labs that do not routinely perform it. PCR is more sensitive and is available through commercial send-out labs, but due to the non-specific nature of acute CD, diagnosis is less likely to be considered outside of highly endemic areas. Microbial cell-free DNA (mcfDNA) testing, such as the Karius Spectrum® test, is a noninvasive diagnostic modality with broad pathogen detection. Karius may be particularly valuable when the diagnosis is not suspected or when infection is suspected but there are delays or barriers to traditional testing. Here we report five cases—one child and four adults—of CD diagnosed by microbial cell-free DNA testing.

**Figure 1.**
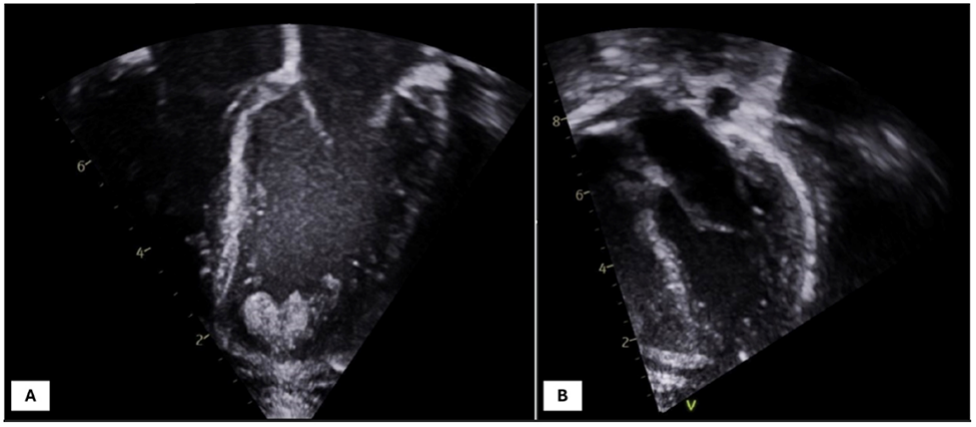
Echocardiogram of a 12-month-old male showing thrombus. A: Thrombus in the left ventricle apex measuring 6x7 mm and 6x6 mm, bilobed or two adjacent clots. Small to moderate pericardial effusion. B: Resolution of left ventricle thrombus.

**Figure 2.**
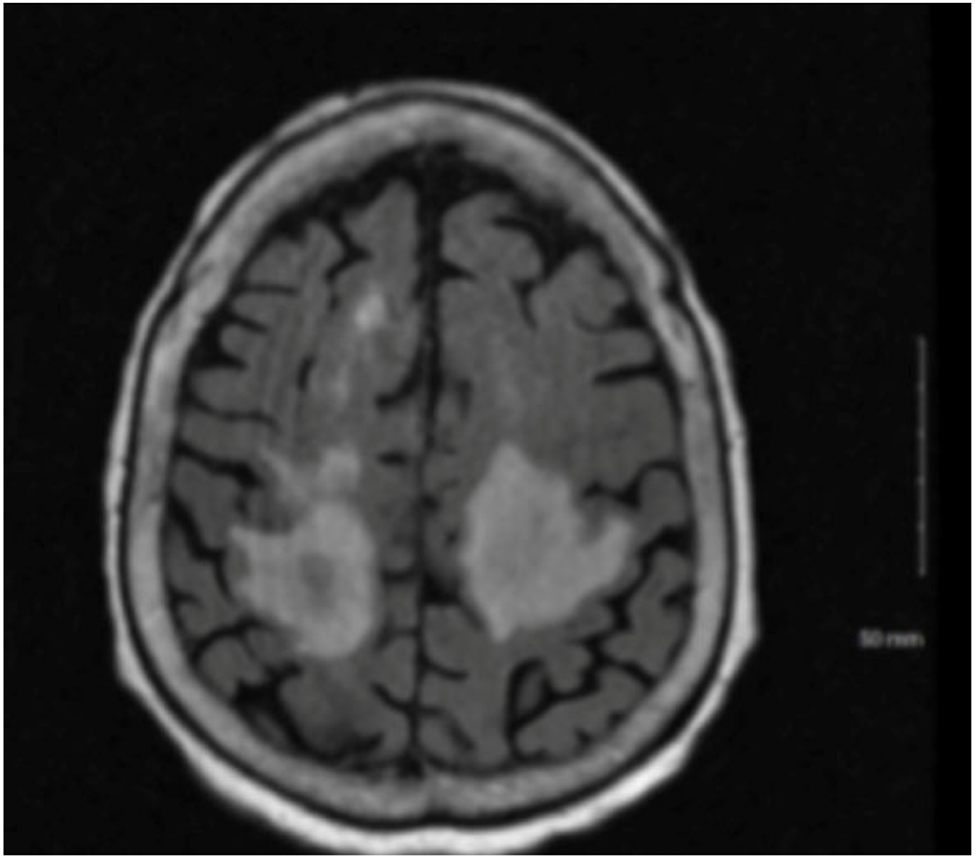
Brain MRI of an 88-year-old female with necrotizing encephalitis demonstrating bilateral frontoparietal enhancing white matter changes and surrounding vasogenic edema.

**Methods:**

We collected clinical, laboratory and demographic information on patients diagnosed with CD using the Karius Spectrum® test, turnaround time, and impact on the management of the patients at four institutions: Driscoll Children’s Hospital, Dell Medical Center, Froedtert Hospital, and UCLA Medical Center.

**Figure 3.**
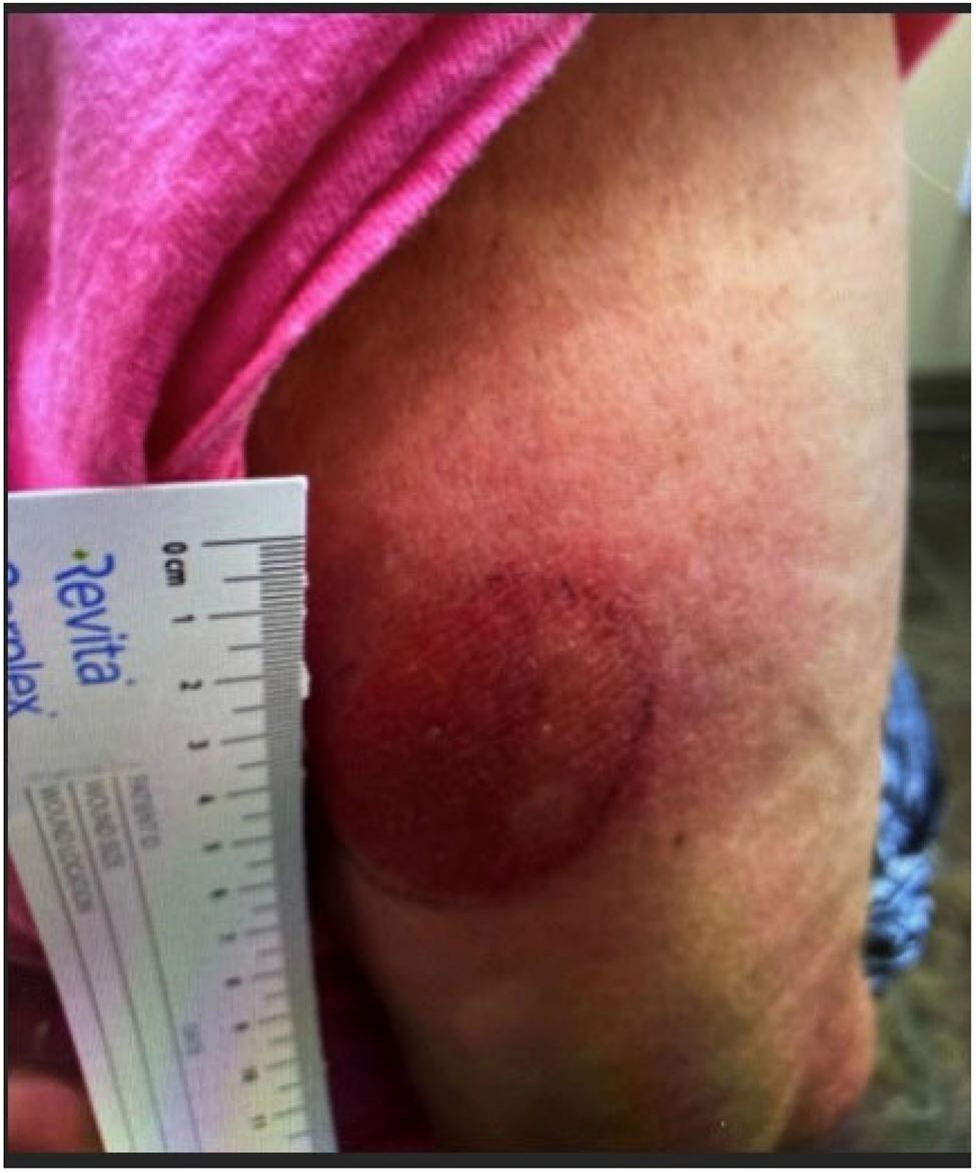
Chagoma on upper right arm of a 71-year-old female presenting as a raised, circular erythematous lesion (∼5 cm).

**Figure 4.**
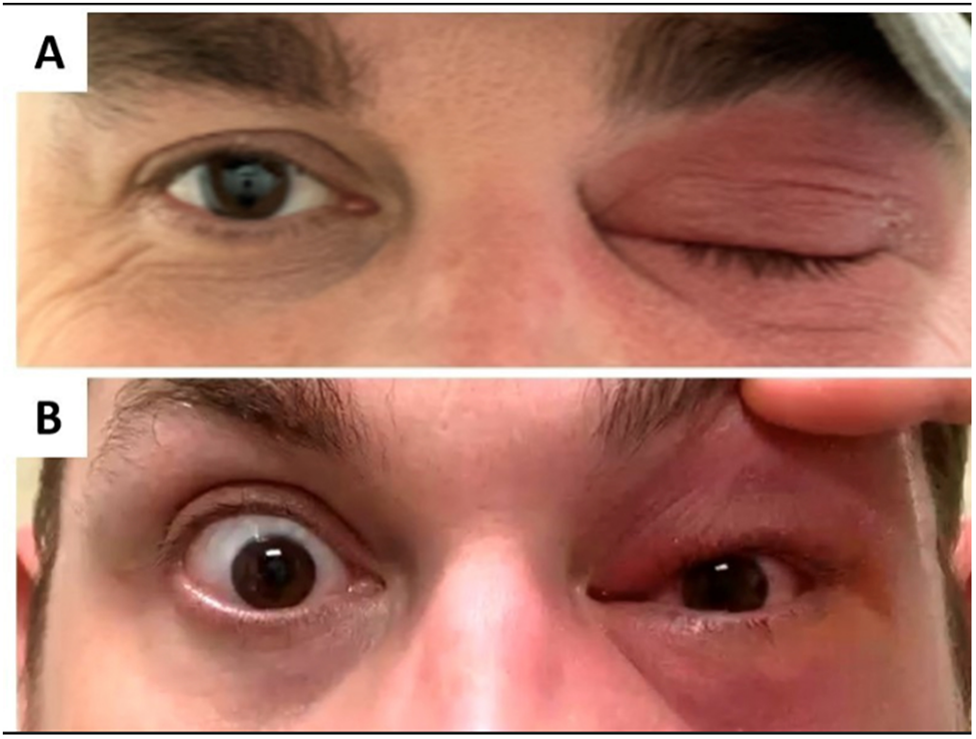
41-year-old male with Romana’s sign on left eye.

**Results:**

Five patients (4 adults, 1 child) tested positive for *T. cruzi* by the Karius Spectrum® test; diagnosis was not initially suspected in four. One case involved suspected Chagas reactivation. Patients (2 male, 3 female; ages 12 months–88 years) presented with fever and variable symptoms. Four were started on empiric therapy before results. Test turnaround was 2–5 days. Three improved after treatment; two expired. Case 1: 12-month-old male with cardiomyopathy (Figure 1). Case 2: 88-year-old female with necrotizing encephalitis (Figure 2). Case 3: 71-year-old female with Chagoma and cellulitis (Figure 3). Case 4: 41-year-old male with orbital cellulitis (Figure 4). Case 5: 50-year-old female with reactivation of known CD.

**Conclusion:**

The detection of microbial cell free DNA in blood can rapidly confirm the diagnosis of Chagas disease and it is a particularly valuable test when the diagnosis is not clinically suspected.

**Disclosures:**

All Authors: No reported disclosures

